# Cohort Profile: The Zurich Project on the Social Development from Childhood to Adulthood (z-proso)

**DOI:** 10.1007/s40865-022-00195-x

**Published:** 2022-02-21

**Authors:** Denis Ribeaud, Aja Murray, Lilly Shanahan, Michael J. Shanahan, Manuel Eisner

**Affiliations:** 1 Jacobs Center for Productive Youth Development, University of Zurich, Zurich, Switzerland; 2 Department of Psychology, University of Edinburgh, Edinburgh, UK; 3 Department of Psychology, University of Zurich, Zurich, Switzerland; 4 Department of Sociology, University of Zurich, Zurich, Switzerland; 5 Violence Research Center, Institute of Criminology, University of Cambridge, Cambridge, UK

**Keywords:** Developmental Criminology, Developmental Psychology, Psychopathology, Juvenile Delinquency, Field Experiment, RCT, Victimisation, Aggressive Behaviour, Violence, Substance Use, ADHD, Socio-Emotional Skills, Risk and Protective Factors, Parenting, Moral Development, Legal Socialization

## Abstract

The Zurich Project on the Social Development from Childhood to Adulthood (z-proso) began in 2004 in response to the need for a better evidence base to support optimal child social development and prevent crime and violence. Since then, the study has tracked the development of a diverse sample of youths (*N* = 1,675 in the target sample; ~50% female) from age 7 (*n* = 1,360) to age 20 (*n* = 1,180), with primary data collection waves at ages 7, 8, 9, 10, 11, 12, 13, 15, 17, and 20. The study uses a multi-method, multi-informant design that combines teacher, youth, and parent reports with observational and behavioural measures, biosampling, functional imaging, and ecological momentary assessment. Analyses of the data have contributed important evidence to a diversity of topics in child and adolescent development, illuminating the developmental roots of crime and aggression, the impacts of exposure to different forms and combinations of victimisation, and trajectories of mental health and neurodevelopmental symptoms.

## Why Was the Cohort Set Up?

The Zurich Project on the Social Development from Childhood to Adulthood (z-proso) was launched in 2004 as a combined randomised field experiment and cohort study. Its primary aims are to contribute to evidence-based developmental violence prevention and to advance understanding of the life-course development of social skills and antisocial behaviour.

Initial ideas for the z-proso study took shape in 2001 when Zurich, Switzerland’s largest city (pop. ~400,000) and amongst the most affluent cities worldwide, faced increased levels of youth violence in low-income, multi-ethnic neighbourhoods. The Council of the City of Zurich commissioned a study to assess levels of violence and preventive structures and resources in Zurich. The final report identified a lack of universal early prevention programmes for families and schools as a major gap in the portfolio of violence prevention in the City of Zurich (Eisner et al., 2003). It recommended a long-term cohort study to examine intervention outcomes and related dynamics in child and adolescent behaviour. Two interventions, each with a strong international evidence base, were identified in subsequent consultations, namely the Triple P-Positive Parenting Program (Sanders, 1999) and the school-based socio-emotional skill intervention Promoting Alternative Thinking Strategies (PATHS) (Kusché & Greenberg, 1994).

Based on this report, a research proposal for a three-wave cohort study, combined with a randomised, controlled effectiveness trial, was submitted to the Swiss National Science Foundation in December 2002 and was approved in March 2003. The proposal laid the foundations for a partnership between two of the co-authors, Manuel Eisner and Denis Ribeaud, and a group of violence prevention experts from the municipality of Zurich. The research team was responsible for the multi-informant cohort study and the scientific design of the experiment. The violence prevention team of the City of Zurich was responsible for delivering the interventions to high standards.

The original purpose of z-proso was fourfold: (1) to describe the social development of children from childhood to adolescence in a diverse urban sample, with a particular focus on aggressive and non-aggressive conduct problems; (2) to contribute to knowledge on the developmental risk factors of aggressive and violent behaviour and the consequences of victimisation; (3) to identify and examine protective factors that could help to strengthen violence prevention policies in families, schools, and neighbourhoods; and (4) to evaluate the short- and long-term effectiveness of two evidence-based universal interventions, delivered through the school system by specialist implementation teams.

## Who Is in the Cohort?

**Fig. 1:**
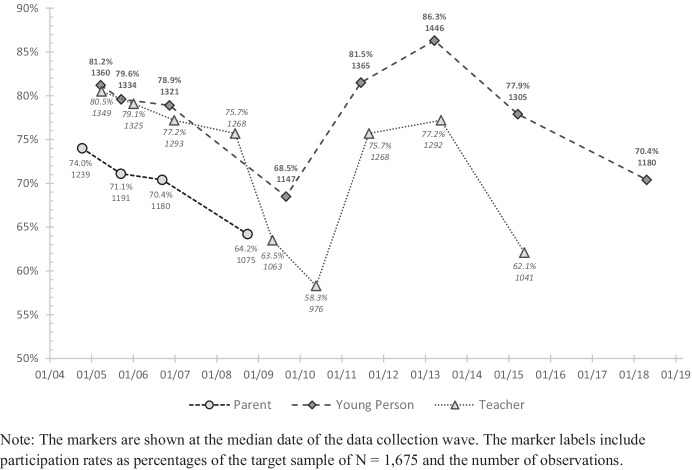
Study participation rates, Jan 2004–Dec 2018. Note: The markers are shown at the median date of the data collection wave. The marker labels include participation rates as percentages of the target sample of *N* = 1,675 and the number of observations

The baseline target population of the study consisted of 2,514 children who entered the first year of school in one of 90 public primary schools in the city of Zurich in August 2004. The sampling procedure was contingent on the cluster-randomised 2*2 factorial design with four experimental conditions (Triple P, PATHS, Triple P*PATHS, and control) and schools as sampling units. Specifically, schools were stratified by small vs. large school size and seven school districts. Within each stratum, four schools were randomly selected and allocated to the experimental conditions. The large school stratum of the most affluent school district was replaced by four day-care schools scattered across the city. Hence, the eligible sample consisted of 14 quadruplets of similar schools, in terms of size and location (except for day-care schools), with 116 classes, comprising 1,675 first graders. Overall, the sampling procedure resulted in a desired, slight overrepresentation of low socio-economic status school districts in the sample. Figure 1 provides an overview of participation rates relative to the initial target sample of 1,675 participants across the data collections as of 2021.

All 56 selected schools took part in the study. In 28 schools, parents were invited to participate in the Triple P parenting programme at Grade 1 (2004/5; Eisner & Meidert, 2011), whereas the PATHS social skills training was implemented at Grade 2 (2005/6), also in 28 schools, 14 of which had been assigned to the Triple P condition at Grade 1. Consequently, 14 schools were not assigned to either programme and constituted the control condition. Participation in the PATHS condition was mandatory for Grade 1 teachers of the selected schools, and thus, all children of the corresponding classes were included in the intervention. In contrast, participation in Triple P was voluntary for the parents of the selected schools. Amongst those parents who also participated in the longitudinal study, 25% completed the four Triple P sessions. Low-SES parents with an immigration background were generally less likely to participate (Eisner & Meidert, 2011).

Participants were recruited to the study in two main stages: At the beginning of the study, all parents or other primary caregivers of eligible children obtained information and were then contacted to provide informed consent. Extensive efforts were made to recruit and retain non-German-speaking immigrant-background families (Eisner & Ribeaud, 2007). These efforts included translating all contact materials and questionnaires into nine languages; recruiting and training bilingual interviewers for all major language groups; and consulting with community representatives on possible concerns relating to participation in the study. In addition, the parental informed consent sought at the beginning of the study (age 7) included the possibility of opting out of the parent interview while consenting to the child interview and the teacher assessment. In this initial recruitment phase, 74.0% (*n* = 1,239) of the parents agreed to participate in the first standardised interview, which was offered in 10 languages. Additionally, 4.3% of German-speaking parents and 12.5% of non-German-speaking parents provided consent to the data collection from the child and the teacher only. Accordingly, the overall child participation rate at the first assessment was 81.2% of the target sample (*n* = 1,360). Overall, children and families in low socio-economic status neighbourhoods and children and families of socially disadvantaged, migration backgrounds were less likely to participate in the study in the first stage (Eisner et al., 2019). This socio-demographic bias is more pronounced in the parent interview sample and, owing to the additional recruitment efforts, less so in the child interview sample.

The second main recruitment stage occurred when study participants were at ages 13 and 15. At this stage, the study team obtained permission to re-contact the entire initial eligible target sample of 1,675 adolescents. Participating youths could actively consent to their participation, though parents retained the right to refuse their child’s participation. Owing to this re-recruitment opportunity, 81.6% of the target sample (*n* = 1,365) participated at age 13, and 86.4% (*n* = 1,446) participated at age 15. A comparison between the sample re-recruited at age 13 and the target sample showed almost no differences in response rates by neighbourhood disadvantage or migration background (Eisner et al., 2019), suggesting that the re-recruitment efforts were successful in eliminating the bias from the initial recruitment stage. Given the association of these characteristics with different types of externalising problem behaviours and delinquency, the re-recruitment was also successful in including additional participants at risk for behavioural problems. However, despite extensive recruitment efforts, consequent data collections at ages 17 and 20 indicated declining participation rates and re-increasing biases as to the socio-demographic background and risk exposure of the participants.

Across all data collection waves, *n* = 1,583 (94.5%) contributed data to at least one wave via at least one informant. As of 2021, the study still has contact details for 1,442 participants, or 91% of those who ever participated, who can thus be re-contacted for future data collections.

Table 1 shows some key characteristics of the initial sample based on the information from the first parent interview, which was conducted with the primary caregiver of the child, usually the biological or adoptive mother (93.9%), but in some instances the father (5.2%), or other interviewee (e.g., foster or step-parent; 0.9%). The sample was highly diverse with respect to the countries of origin and native languages of the parents. A majority of the participants’ mothers (58%) were not born in Switzerland. Fifty-three percent of primary caregivers in the target sample were not native German speakers, with Albanian, Bosnian/Croatian/Serbian, Portuguese, Turkish, Spanish, Tamil, and Italian being the largest language groups (Eisner & Ribeaud, 2007). This diversity reflects the substantial proportion of second-generation immigrant children and adolescents amongst young people in Swiss cities and in Zurich in particular (Topgül et al., 2015). The most common country of origin of immigrated parents in the sample was former Yugoslavia. Many of those parents arrived in Switzerland in the wake of the civil wars from 1991 to 2001. Other common countries of origin (>2%) included Germany, Portugal, Sri Lanka, Turkey, and Italy. The family backgrounds of study participants also varied widely with respect to parental education and socio-economic status.

**Table 1:** Selected sample characteristics (*n* = 1,239), based on data from the first parent interview in 2004/5 at child age 7

*Child characteristics*	
Month of birth *(M/SD)*	Oct 1997/4.43 mo.
Gender *(% boys)*	51.9%
Birth country *(% Switzerland)*	89.6%
*Female primary caregiver year of birth (M/SD)*	1967/5.37 y.
*Female primary caregiver maternal language not German*	55.5%
*Female primary caregiver country of birth*	
Switzerland	42.3%
Former Yugoslavia	14.8%
Germany	5.7%
Portugal	4.8%
Sri Lanka	4.1%
Turkey	3.9%
Italy	2.4%
Other countries (with proportions <2%)	22.0%
*Female primary caregiver education*	
Low (9 or less years of compulsory school plus max. 1 year of elementary vocational training)	25.4%
Medium (e.g., full vocational education and grammar/high school)	58.4%
High (university degree or equivalent)	16.0%
*Household characteristics*	
Parent structure *(% both biol. parents)*	77.8%
Number of children in HH *(M/SD)*	2.16/0.92
Unemployment *(% either parent)*	7.8%

## How and How Often Have They Been Followed Up

To date, ten main data collection waves have been realised at ages 7, 8, 9, 10, 11, 12, 13, 15, 17, and 20 and resulted in 30 primary data collection points.

Table 2 provides an overview of the data collections.

**Table 2:** Main data collection overview, z-proso, and associated studies

Module	Ages	Themes (selection)
*(A) Primary Data Points*		
Parent interviews	7, 8, 9, 11	Demographic screen, SBQ, APQ, DAS-7, GHQ, migration history, family climate, life events
Child face-to-face interviews	7, 8, 9	SBQ, social problem-solving, emotion recognition, bullying
Young person questionnaires and assessments	11, 13, 15, 17, 20	SBQ, APQ (adapted), delinquency, substance use, self-control, routine activities, life events, biceps circumference, resting heart rate
Teacher assessments (plus complementary vocational trainer assessment at age 17)	7, 7.5, 8, 8.5, 9, 10, 11, 12, 13, 15, 17	SBQ, social role, academic achievement, school climate
Interviewer assessments, child interview	7, 8, 9	Cooperation, restlessness, language skills
Interviewer assessments, parent interview	7, 8, 9, 11	Interview setting, language skills, residential environment
*(B) Intervention* *Assessment*		
PATHS observational assessments of curriculum delivery	8/9 (Four assessments during year 2 of primary school)	Intervention fidelity, teacher enthusiasm, student engagement
PATHS teacher progress form on lessons delivered	8/9 (Bi-weekly forms)	Time on PATHS lessons, lessons delivered
Triple P facilitator fidelity assessments, participant satisfaction questionnaire	7/8 (Weekly attendance sheets, facilitator assessments)	Parent attendance, group engagement, coverage of curriculum contents
*(C) Administrative Data* *Collections*		
Neighbourhood characteristics	3, 8	Census area scores on familism, socio-economic status, ethnic heterogeneity, survey on neighbourhood cohesion
Educational career	7, 11, 13, 15, 17	Special needs school, retention, school track, type of apprenticeship
Youth justice contact	10–17	Youth justice record (canton of Zurich residents only), type of main offence, associated offences, type of judicial decision and sanction
*(D) Associated* *Studies*		
*Decades-to-Minutes Ecological Momentary Assessment* (PI: Aja Murray, University of Edinburgh)	20 (Subsample only, two weeks, 4 prompts per day)	Activities and context, emotional state, aggression, provocations, substance use, stress
*Stress Responsivity Study z-GIG* (PIs: Todd Hare & Michael Shanahan, University of Zurich)	20	Eye tracking, brain activity during decision-making games, immune gene expression
*Hair Sample Study* (PIs: Lilly Shanahan and Boris Quednow, University of Zurich)	20	Hair cortisol, cortisone, testosterone, metabolites of psychoactive drugs and medication
*COVID-19 Well-Being Study* (PIs: Manuel Eisner, Denis Ribeaud, Lilly Shanahan, Urs Hepp, University of Zurich)	22 (four waves, May–Sept 2020)	Pandemic-specific strains, emotional distress, substance use, stress, coping, COVID-19 rule compliance

The parent or other primary caregiver was interviewed at home for about 60 minutes via computer-aided personal interviewing (CAPI) when the target child was 7, 8, 9, and 11 years old. The children were assessed at ages 7, 8, and 9 via CAPI on the school premises (45 minutes). Interviewers completed brief assessments of the interview situation and the interviewee for each child and parent interview.

Teacher reports on participants, including complementary reports from vocational trainers at age 17, were collected 11 times between participant ages 7 and 17 using a paper-and-pencil questionnaire. Teachers also provided school-wide assessments on the school climate and the provision of prevention programmes in their school.

At ages 11, 13, 15, and 17, the participants were assessed by trained fieldworkers in group settings on the school premises using standardised paper questionnaires. At age 20, participants were invited to a social science research laboratory in a central location in Zurich for computer-assisted self-completed interviews.

During the 2020 lockdown measures due to the COVID-19 pandemic, study participants were invited to take part in four waves of supplementary online assessments between April and September 2020 (Nivette et al., 2021b; Shanahan et al., 2020).

During the first years of the study, data were also collected to document intervention delivery and integrity. Teachers in the PATHS intervention condition contributed assessments of the training provision and returned weekly summaries of the programme elements delivered. Up to four lessons in each class were attended by PATHS facilitators, who assessed the quality of the delivery via standardised assessment tools.

In 2015, 1,274 participants (97.5% of those eligible) consented to a full search of their youth criminal record data, which were collected and coded from 2017 to 2021.

Informed consent from the parents and/or youths was obtained in accordance with the relevant national regulations, and all data were processed and stored according to data protection regulations. Children, for whom parents provided informed consent at ages 7 to 11 and who themselves did not wish to participate, were free to decline participation. Given the minimally intrusive nature of the study design, questions, and intervention, ethical approval was not initially required in accordance with Swiss regulations. Since 2017, ethics approval for the main study has been provided by the Ethics Committee at the Faculty of Arts and Social Sciences of the University of Zurich. Separate approvals were obtained for add-on studies led by collaborating research teams (see below).

## What Has Been Measured?

With regard to the key outcome variables, the study has continuously focused both on the life-time development of different types of externalising and internalising problem behaviours and on violent victimisation. At the onset, the study targeted two overarching goals that drove the selection of the research instruments. The first goal was to assess the quality of implementation (process evaluation) and the effectiveness (outcome evaluation) of the two prevention programmes. The second goal was to identify, on the basis of a social-ecological approach, risk factors of problem behaviour and violent victimisation in key risk domains (individual, family, school, and neighbourhood), as well as mechanisms involved in the development of such behaviour and experiences. Whereas a key set of scales has been consistently delivered across all, or most, data collections, measurement scales were furthermore included and excluded in accordance with the participants’ evolution through different life stages. A collaborative work with research teams from other disciplines was another important source for the inclusion of novel scales.

The z-proso research team has adopted a multi-informant and multi-method approach to data collection. For most participants, five different types of informants (focal study participant, primary caregiver, teacher, child interviewer, and parent interviewer) contributed data at repeated assessment points. In addition, z-proso comprises various kinds of matched administrative and population survey data. Types of data used in z-proso include data from standardised questionnaires, scenario-based techniques, event-history calendars, experiments, official records, and ecological momentary assessment, as well as observational data, qualitative data, and biological data, including anthropometric data, hair samples, fMRI imaging data, and whole blood.

A core element of the parent interviews at child ages 7 to 11 was the repeated assessment of child social behaviour using the Social Behaviour Questionnaire (SBQ), an omnibus instrument that covers prosocial behaviour, aggressive and non-aggressive behaviour problems, internalising symptoms, and symptoms of attention-deficit/hyperactivity disorder (Murray et al., 2019b; Tremblay et al., 1991). Repeated assessments also included the Alabama Parenting Questionnaire (Shelton et al., 1996), which assessed parenting behaviour and a brief family climate scale. Event history calendars provided information about life events since the birth of the child, including parental separation, unemployment, and major illnesses (Eisner et al., 2009). Other topics covered included household composition and socio-economic status, substance use during pregnancy, perinatal complications, migration history and acculturation of families with a migration background, general well-being of the primary caregiver, utilisation of parenting support, family functioning, and behaviour problems of siblings of the child (Ribeaud & Eisner, 2010b).

The teacher assessments at participant ages 7 to 17 measured dimensions of child social behaviour using the SBQ (Tremblay et al., 1991). They also included assessments of school achievement and motivation, as well as the role of the child in the classroom. Additionally, teachers completed brief assessments on classroom and school characteristics, such as class cohesion, school-level disruptive and antisocial behaviours, and teacher cooperation.

Multiple informants provided evaluated the implementation of the interventions. For the school-based PATHS programme, facilitators attended lessons and assessed implementation fidelity, teachers provided satisfaction ratings of the PATHS training and bi-weekly data on delivered lessons, and children were asked about their knowledge of intervention-related concepts and activities. For the parenting intervention Triple P, facilitators returned attendance sheets and assessments of parental engagement during the workshops, a member of the research team observed a sample of programme sessions, and parents assessed satisfaction with the Triple P programme and reported utilisation of programme-related techniques (Eisner & Meidert, 2011).

In the first years of primary school, repeat measures used in the child interviews included a newly developed child multimedia version of the SBQ; social problem-solving scenarios to assess changes in social problem-solving skills (Dodge & Coie, 1987); and emotion recognition tasks (Schultz et al., 2004). Additional instruments measured, amongst other variables, individual characteristics such as sensation seeking (Alsaker et al., 2008), impulsivity, and risk taking; bullying experiences; the child’s socio-metric status in the class; interests and preferred activities; and attribution of moral emotion (Nunner-Winkler & Sodian, 1988). Interviewers provided standardised assessments of child behaviour during the interview.

Many instruments used in the young person assessments at age 11 were administered, with age-appropriate modifications, in all waves to age 20. For example, respondents provided information in all waves about bullying victimisation and perpetration, the social behaviour domains covered in the SBQ, leisure activities at home and outside the home, media use (including problematic media use), self-control, conflict coping strategies, and information on best friends and their behaviour. Respondents also provided self-reported data on delinquency and substance use. By age 13, these scales were extended, and questions were added on various aspects of contact with the police, including whether the respondent had had such contact as perpetrator, witness, or victim and what the consequences of the contact were.

Some constructs that had been assessed in the parent interviews at ages 7 to 11 continued to be measured as self-reports. For example, an adapted self-report version of the Alabama Parenting Questionnaire was administered until age 17, and a life events questionnaire administered from age 13 to 20 continued to assess aspects of the event history calendar. Various repeat instruments were administered to measure traits, cognitions, and emotions related to violence, as well as processes of moral and legal socialisation. Measures include aggressive decision-making scenarios, measures of legal cynicism and police legitimacy, moral neutralisation of violence, guilt, shame, and norms of masculinity. Some measures were aimed at capturing age-specific developments. For example, a pubertal development scale was administered at ages 13 and 15, while instruments administered at ages 17 and 20 measured aspects such as sexual experiences, dating violence, and the purchase or sale of sexual services.

Starting at age 13, data on physical constitution and health were collected, initially including body mass index and pubertal development. Data on participants’ biceps circumference, resting heart rate, self-assessed physical attractiveness, and fighting ability were collected at ages 15, 17, and 20.

Various types of administrative data have been linked to the data collected from participants. Data from the education authorities of the City and Canton of Zurich provide information about the educational trajectories of the study participants at ages 7, 11, 13, 15, and 17. Census data for the year 2000 provide information on neighbourhood characteristics, such as social class, residential stability, and ethnic heterogeneity. In addition, for participants with a migration background, linked administrative data provide information on country-level characteristics, such as gender inequality, GDP per capita, and violent conflict. Finally, full criminal record data, including police and youth justice files, are included from ages 10 to under 18.

At age 20, several associated studies were realised by collaborating teams. Heiko Rauhut and his team at the University of Zurich adapted a set of decision-making games (honesty game, trust game, public good game, dictator game, and ultimatum game) for the age 20 CASI survey. Lilly Shanahan and Boris Quednow (University of Zurich) collected hair samples from 1,003 study participants (85% of the age 20 sample). Cortisol/cortisone, testosterone, and metabolites of substances and medications were quantitatively determined by liquid chromatography–tandem mass spectrometry (LC–MS/MS) at the Institute for Forensic Medicine at the University of Zurich (Scholz et al., 2020). For a subsample of *n* = 200 participants at age 20, a research team led by Todd Hare and Michael Shanahan (University of Zurich) collected eye-tracking data from behavioural tasks to assess stress thresholds and reactivity; n = 122 of those also included fMRI measures of brain function. For this subsample—composed of participants who were pre-selected by coarsened exact matching to examine effects of victimisation by bullying—the team also collected mRNA sequencing data, DNA, and a cytokine panel, all based on peripheral blood draws. For another collaborative study, led by Aja Murray (University of Edinburgh), *n* = 255 z-proso participants took part in an ecological momentary assessment following the age 20 assessment. Participants were prompted four times per day over two weeks to report on sensations such as momentary aggression, provocation, and affective state (Murray et al., 2020d, 2021b, d).

## What Has Been Found?

To date, data obtained from the z-proso study have led to one monograph and more than 90 peer-reviewed journal articles in fields such as criminology, prevention science, developmental psychopathology, epidemiology, psychiatry, psychometrics, and public health (https://www.jacobscenter.uzh.ch/en/research/zproso/aboutus/publication_list.html). The z-proso International Research Network (zIReN) promotes collaborative research based on z-proso data. It currently includes over 30 researchers at 14 universities worldwide and has been supported by the Jacobs Center for Productive Youth Development, University of Zurich.

### Intervention Effects

Several studies have examined the delivery and effects of the two interventions. Intention-to-treat analyses up to age 11 suggested no significant effects of the parenting programme Triple P on parenting, social skills, or symptoms of externalising and a positive, significant effect of the social skills programme PATHS on teacher-assessed externalising behaviours at age 11 (Malti et al., 2011). A follow-up study examined the effects of the interventions on adolescent delinquency and substance use at ages 13 and 15 based on self-reports and teacher reports (Averdijk et al., 2016b). It found mostly non-significant effects. Moreover, these two studies did generally not find significant beneficial effects for the combined Triple P and PATHS treatment.

Other studies have examined factors associated with participation in the parenting programme and treatment effects on parenting and child behaviour in the subgroup of highly compliant parents (Eisner et al., 2012; Eisner & Meidert, 2011). Up to age 13, no consistent effects on either five dimensions of parenting practices or five dimensions of child problem behaviour were found.

### Longitudinal Cohort Methodology

Several studies have examined methodological concerns regarding cohort studies, including attrition, instrument validity, and cross-sectional and longitudinal cross-informant agreement. Eisner and Ribeaud (2007) analysed the success of recruitment and retention efforts in the z-proso sample and showed that additional recruitment efforts were important because they resulted in the inclusion of children with higher levels of behaviour problems. Another study described the non-monotonic attrition patterns from ages 7 through 17 and analysed differential attrition by behaviour and social background characteristics and found that low education and having a non-German-speaking parent were predictive of attrition (Eisner et al., 2019).

Various studies have presented the psychometric qualities of new instruments developed for z-proso. These include the z-proso Event History Calendar (Eisner et al., 2009), the Moral Neutralisation of Aggression Scale (Ribeaud & Eisner, 2010a), the Zurich Brief Bullying Scale (Murray et al., 2021a), the Violent Ideations Scale (Murray et al., 2018b), and the violent extremist attitudes scale (Nivette et al., 2017).

The psychometric properties of the SBQ (Tremblay et al., 1991), the main instrument for assessing the psychosocial health of the z-proso participants, have also been assessed in various studies. These studies have shown that the SBQ is a valid transdiagnostic tool for a dimensional assessment of symptoms of psychopathology from childhood to adolescence (Murray et al., 2019b, c). Other work based on the SBQ has contributed to knowledge on cross-informant agreement in child behaviour (Murray et al., 2018a).

### Developmental Risk Domains and the Role of Life Events

z-proso generated a large range of observational studies based on the full sample, including all treatment groups of the field experiment. Several studies have examined developmental risks for aggressive behaviour and related externalising symptoms within a socio-ecological framework. In particular, Ribeaud and Eisner (2010b) comprehensively assessed various risk domains associated with aggressive behaviour in early adolescence. They found that proximal internal risk factors were more strongly associated with aggression than distal external risk factors, that the evidence in z-proso was consistent with a cumulative risk model, and that boys showed a greater vulnerability to developmental risk factors than girls.

Several studies have specifically focussed on the role of various life events and experiences, such as early childcare (Averdijk et al., 2011), parental separation (Averdijk et al., 2012), placement in foster care (Averdijk et al., 2018), and quality of relationships with teachers during primary school (Obsuth et al., 2017, 2021). For example, using the event history calendar, Averdijk et al. (2012) showed that parental separation negatively affected child behaviour problems both directly and indirectly via effects on maternal depressive symptoms. Obsuth et al. (2017) used propensity score matching to examine how differences in teacher–child relationship quality affected short- and long-term problem behaviour. They showed that an improved teacher–child relationship in late childhood was associated with fewer short-term behaviour problems and a lower risk of long-term engagement in delinquent acts up to age 17.

### Co-Occurring Mental Health Issues and Developmental Cascades

The z-proso study includes comprehensive dimensional and transdiagnostic multi-informant measures of psychopathology from ages 7 to 20. Based on these data, several studies have contributed to knowledge on developmental psychopathology, with a focus on developmental patterns of co-occurring mental health issues, developmental cascades, and sex and age differences in the onset of symptoms of poor psychosocial functioning. For example, research on the co-occurrence of mental health issues across multiple dimensions confirmed a general tendency towards substantial shared variance across a wide range of mental health issues (e.g., anxiety, ADHD, and aggression), known as the ‘p-factor’ (Caspi et al., 2014), and found that its strength remained relatively constant from ages 7 to 15, in contrast to both the differentiation and dynamic mutualism theories of the p-factor (Murray et al., 2016).

Subsequent research examined developmental cascades across domains of poor psychosocial functioning and found evidence of heterotypic continuity insofar as ADHD symptoms tended to predict both aggressive and non-aggressive symptoms later in life (Obsuth et al., 2020). Moreover, z-proso contributed the first within-person analyses of developmental cascades between internalising problems and externalising problems and found evidence of externalising-to-internalising cascades until adolescence (Murray et al., 2020c). Reciprocal cascades have also been examined in other domains, such as those between ADHD symptoms and anxiety (Murray et al., 2020a) and bullying perpetration (Murray et al., 2021e) in adolescence, as have mediators of cascades, such as peer and teacher relationship problems (Murray et al., 2021c). This evidence illustrated (reciprocal) cascades in multiple connected domains and highlighted the need for more comprehensive developmental cascade models that capture this inter-relatedness and pay greater attention to the linking mechanisms.

Other studies have examined long-term trajectories of ADHD symptoms and their predictors and associated outcomes and have found additional support for a late-onset (i.e., adolescence) trajectory of ADHD symptoms (Murray et al., 2019a) and emerging evidence that childhood sensation seeking may predict later onset trajectories amongst those who initially show no evidence of elevated symptoms (Murray et al., 2020b).

In relation to the 2020 pandemic, a study found that pre-pandemic distress and during-pandemic lifestyle and economic disruptions were strongly associated with distress during the pandemic but that certain coping strategies (e.g., keeping a daily routine, physical activity, and positive reframing) during the first Swiss lockdown were associated with reducing distress (Shanahan et al., 2020).

### Internalising Symptoms, Self-Harm, and Suicidality

Several z-proso studies have specifically examined internalising symptoms, self-harm, and suicidality, with a focus on the transition from early adolescence to early adulthood. One study showed that 27% of young people reported self-harm at least once between ages 13 and 20 (Steinhoff et al., 2020). Precursors of recurrent self-harm included early internalising symptoms and early self-injury onset (Steinhoff et al., 2021). The risk of self-harm increased with an increasing number of life events (Steinhoff et al., 2020). Less than half of the adolescents with self-injury contacted mental health services (Steinhoff et al., 2021).

### Victimisation from Childhood to Early Adulthood

Repeat measures of victimisation at the family, peer, dating partner, and community levels from childhood to early adulthood have facilitated studies on predictors and consequences of victimisation, including studies on the concurrent and dynamic links between victimisation and perpetration. Zych et al. (2020), for example, identified patterns of change and stability in bullying roles at ages 11, 13, 15, and 17 using latent transition analysis. In addition to developmental stability, the analyses revealed a substantial level of change in bullying roles, although transitions from a bully-only role to a victim-only role were rare.

Another study (Averdijk et al., 2016a) examined how violent victimisation in early adolescence may affect decision-making processes associated with a greater probability of violent offending in mid-adolescence. It was found that victimization affects appraisal processes linked to conflict situations. Victims are more likely to anticipate positive feelings about violent offending while experiencing reduced feelings of anticipated shame.

Similarly, one study examined the association between poly-victimisation and subsequent violent ideations in late adolescence and early adulthood. It found an exposure–response relationship insofar as each additional victimisation experience added to the probability of an individual experiencing ideations of inflicting harm on another person (Eisner et al., 2021).

### Legal Socialisation, Moral Development, and Violent Extremism

Several studies have examined the dynamics of moral and legal socialisation from childhood to early adulthood, including extremist attitudes and beliefs. One study showed that legal cynicism in mid-adolescence is strongly predicted by antecedent delinquent behaviour. This finding suggests that legal cynicism may best be understood as a cognitive restructuring mechanism that morally neutralises antisocial harm-doing against others (Nivette et al., 2015). Another study examined developmental antecedents of violent extremist attitudes at age 17 (Nivette et al., 2017), showing that young people who already endorsed general justifications for violence and rule-breaking at an earlier age were more likely to espouse extremist violent views, especially if their families had experienced collective social strife, conflict, or repression. A recent article contributed to the limited literature on within-individual change in extremist beliefs between late adolescence and early adulthood. It was demonstrated that increased self-control, more prosocial peers, and better conflict resolution skills were associated with a decline in violent extremist attitudes (Nivette et al., 2021a).

## What Are the Main Strengths and Weaknesses?

One of very few long-term cohort studies in Europe with a focus on delinquency and violence, z-proso includes a wide range of measures across important dimensions of psychopathology. It comprises a large representative sample of participants, with a high proportion of young people with migration backgrounds. Accordingly, the sample is culturally and socio-economically very diverse and representative of many larger cosmopolitan European cities. Moreover, the study consists of a dense sequence of assessments from childhood to early adulthood and has achieved high recruitment and retention rates. It includes data from multiple informants, biological data, and official records from school and justice authorities. It is also one of very few cohort studies to include a randomised intervention component with the potential to examine the long-term effects of childhood interventions into early adulthood. As well as using established instruments, the study implemented and validated a range of innovative measures that expand our understanding of psychosocial development from childhood to early adulthood.

Nevertheless, researchers interested in analysing the z-proso study should be aware of some limitations. First, while over 70% of the initial target sample were still participating in the study at age 20, attrition was disproportionately high amongst young people with low education and migration background. Also, attrition was higher for participants with higher levels of problem behaviour (Eisner et al., 2019). Second, the cohort is representative of young people growing up in an urban environment in one of the most affluent cities in the world. It is thus not representative of young adults in Switzerland in general nor is it generalizable to strongly underprivileged, poorly developed urban contexts. Third, the study currently has a limited programme of biological data. There is no detailed biobank or linkage to the medical records of the study participants. Researchers should also note that that the sample size makes separate analyses on very rare outcomes (e.g., sexual assault) or specific demographic subgroups (e.g., distinct groups of migrants and specific sexual orientations) difficult. In addition, records of criminal offending are limited to the youth justice system (ages 10–17) and to residents of the canton of Zurich. However, these records include all offences committed in Switzerland by study participants, provided they still live in Zurich, which was the case for 95% of the participants at age 17.

## Can I Get Hold of the Data? Where Can I Find Out More?

Further information on the study can be found on the website of the z-proso Research Infrastructure (https://www.jacobscenter.uzh.ch/en/research/zproso/aboutus.html). Researchers can also obtain additional data documentation from the team at the Jacobs Center for Productive Youth Development at the University of Zurich. This includes an extensive data dictionary and descriptions of all instruments administered as part of the z-proso study.

Substantial parts of the z-proso data will be made available from 2022 on FORSbase (https://forsbase.unil.ch/). We recommend that researchers aiming to use the data consult with the z-proso team at the Jacobs Center for Productive Youth Development.

## Profile In A Nutshell

### Why the Cohort Was Set Up and Unique Features of the Cohort 

Begun in 2004, z-proso responded to the need for a more robust evidence base for preventing problem behaviour. It combines a field experiment to evaluate the effectiveness of two prevention programmes in primary school with a long-term observational panel.

### Location, Year(s) of Baseline Data Collection, Number of Participants at Baseline, and Composition of the Study Population including Age Range

The target population consists of 1,675 children who entered Grade 1 at one of the 56 randomly selected study schools in Zurich in 2004; 1,360 children participated at baseline (age 7), and re-recruitment resulted in participation increasing to 1,446 (86.3%) at age 15 in 2013.

The study population is representative of the city of Zurich’s youth population.

### Frequency of Follow-Up, Attrition, and Number of Participants Currently in the Cohort

Data collections include three school-based CAPI interviews with the target child (ages 7, 8, and 9), followed by four paper-and-pencil surveys in classrooms (ages 11, 13, 15, and 17), and one laboratory-based CASI survey at age 20; four parent CAPI interviews at child ages 7, 8, 9, and 11; and 11 teacher postal surveys at child ages 7 to 17. The latest data collection at age 20 had 1,180 participants (70.4%).

### Main Categories of Data Collected

Parent interviews focus on child social behaviour, parenting, family life events, family conflict, parent psychopathology, service use, and socio-demographics. Child interviews (age 7–9) comprise assessments of social behaviour, conflict resolution skills, emotion recognition, sensation seeking, and popularity. Youth surveys (age 11–20) focus on social behaviour, delinquency, substance use, life events, leisure activities and peer relationships, self-control, and violence-related attitudes and cognitions. Teacher surveys focus on child social behaviour, delinquency, school achievement, and class/school characteristics. Administrative data provide information on school career and recorded crime.

### Collaboration and Data Access

The z-proso International Research Network (zIReN) includes over 30 researchers at 14 universities worldwide. The project data is largely open access and will be made available on FORSbase in 2021. Interested researchers are invited to contact the study directors to join the network and to access the data and documentation.
